# Identification, diversity and domain structure analysis of mucin and mucin-like genes in sea anemone *Actinia tenebrosa*

**DOI:** 10.7717/peerj.13292

**Published:** 2022-05-05

**Authors:** Alaa Haridi

**Affiliations:** School of Biology and Environmental Science, Faculty of Science, Queensland University of Technology, Brisbane, QLD, Australia

**Keywords:** *Actinia tenebrosa*, Cnidaria, Mucus, Gel-forming mucin, Transmembrane mucin, Transcriptomes, Sea anemones, Mucin genes, Assembly, RNA-sequencing

## Abstract

**Background:**

Mucins are part of the glycoprotein family and the main proteinaceous component of mucus. The sea anemone species, *Actinia tenebrosa* (Phylum Cnidaria) produce large amounts of mucus, which have not been studied in detail. Furthermore, there has only been limited investigation of mucin genes in phylum Cnidaria. Therefore, the aim of current study was to identify and analyse the repertoire mucin genes present in *A. tenebrosa* and range of other sea anemone species to document their diversity in this group.

**Methods:**

To achieve this aim, we undertook transcriptome sequencing, assembly, and annotation to identify mucin genes in *A. tenebrosa*.

**Results:**

The results from this study demonstrated a diverse repertoire of mucin proteins, including mucin1-like, mucin4-like, and a range of mucin-like genes in the range of sea anemone species examined. The domain structure of the identified mucin genes was found to be consistent with the conserved domains found in the homologous proteins of vertebrate species. The discovery of a diverse range of mucin genes in sea anemone species provided a basic reference for future mucin studies in cnidarians and could lead to research into their application in the pharmacological, clinical, and cosmetic industries.

## Introduction

Mucins are a part of the glycoprotein family and the main proteinaceous component of mucus (Fahy & Dickey, 2010; Wang et al., 2017). Their general structure and biochemical properties protect cell surfaces, and their specific molecular structures regulate the local molecular microenvironment near the cell surface (Hollingsworth & Swanson, 2004). Additionally, they can also relay information about the condition of the external environment to epithelial cells through signal transduction, which occurs *via* membrane-associated mucins (Chambers et al., 1994). Thus, mucins can also act as a protective barrier; block the passage of bacteria, large molecules, and other infectious organisms (Pelaseyed et al., 2014).

**Figure 1:**
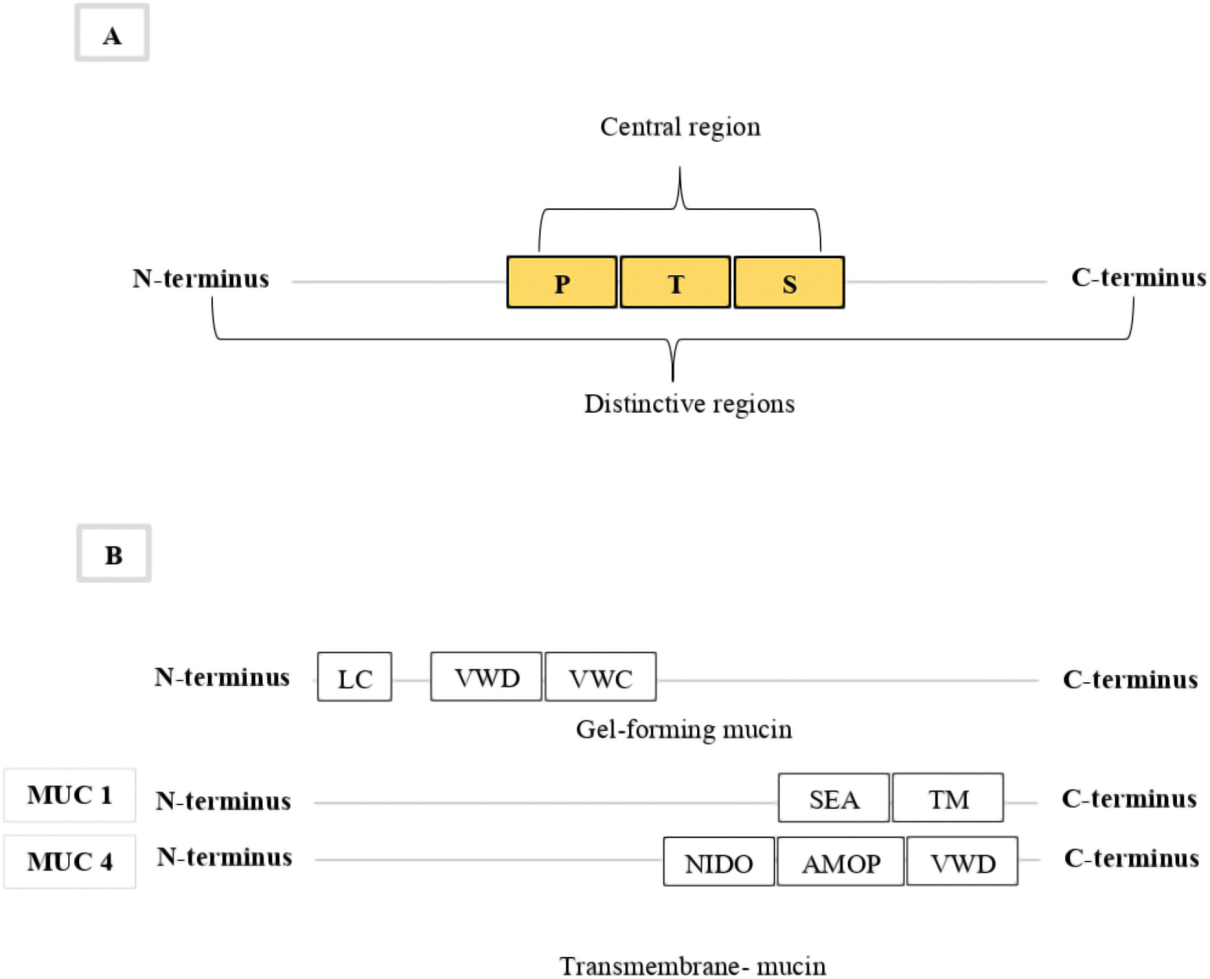
The general structure for mucins. The figure show in (A) general structure for all mucins indicating the main regions; first, the distinctive terminal regions (N and C), and second, the large central region (PTS). (B) Indicating the conserved gel-forming and transmembrane mucin domains use to identify and classify mucin types.

The mucins usually comprise up to 15–20% of the polypeptide component and over 70% of carbohydrates, especially the o-linked glycans, in mucus (Gendler & Spicer, 1995; Offner & Troxler, 2000). A small ratio of N-glycan has also been identified in mucin proteins (Asker et al., 1998; Yu et al., 2008; Kaur et al., 2013). Each mucin glycoprotein has two distinctive regions; first is the amino- and carboxyl-terminus (N and C terminus) regions, and the second is the large central region (Fig. 1A). The amino- and carboxy-terminus regions are very sparsely o-glycosylated, but rich in cysteines. The central region of the mucin structure is characterised by amino acids which are rich in proline (P), threonine (T) and serine (S) residues (Lang, 2007; Keeley & Mecham, 2013; Arike & Hansson, 2016; Malin & Gunnar, 2016). These PTS residues are the hallmark of all mucin proteins (Chaturvedi, Singh & Batra, 2008; Xu et al., 2016) and are made up of more than 40% threonine and serine and about 5% proline residues (Arike & Hansson, 2016). These hallmark PTS residues are highly O-glycosylated, play an important functional role in forming defensive mucus gel (Keeley & Mecham, 2013; Zaretsky & Wreschner, 2013; Arike & Hansson, 2016), and are used to identify mucin protein sequences (Hansson, 2012; Corfield, 2015; Arike & Hansson, 2016). These domains are highly variable tandem repeat sequences and are sometimes also referred to as VNTR domains (Variable numbers of tandemly repeated) (Bythell & Wild, 2011; Arike & Hansson, 2016; Malin & Gunnar, 2016). As a result, mucin genes vary widely in the number of their PTS domains, their length, and the sequence of amino acids in these domains (Gendler & Spicer, 1995; Karakoç, Sağsöz & Ketani, 2016). The PTS/VNTR domains are varied and non-conserved domains across a mucin protein, which make it difficult to use these domains to classify specific mucin types (Gendler & Spicer, 1995; Karakoç, Sağsöz & Ketani, 2016).

The most common molecular classification of mucin protein types is based on domain structures that can be used to categorize mucin proteins into two subgroups: secreted gel-forming and membrane-bound mucins (Desseyn et al., 2000; Coppin et al., 2017). The secreted gel-forming group include: MUC2, MUC5AC, MUC5B, MUC6, and MUC19 (Lee et al., 2016), while the transmembrane group include: MUC1, MUC3A, MUC3B, MUC12, MUC13, MUC16, MUC17 and MUC4 (Zaretsky & Wreschner, 2013). The domain architecture of gel-forming mucins consists of conserved domains containing a signal peptide (SP) in the N-terminus, cysteine-rich von Willebrand factor type D (VWD) and type C (VWC) domains, and a central PTS/VNTR domain (Lang, 2007) (Fig. 1B). The C-terminus of gel-forming mucins includes other cysteine-rich domains and the cysteine knot domain (Zaretsky & Wreschner, 2013). The number of the cysteine-rich domains varies among the gel-forming mucins (Zaretsky & Wreschner, 2013). The domain architecture of transmembrane mucins also consists of conserved domains containing a SP in the N-terminus and a central PTS/VNTR domain (Lang, 2007). The C-terminus of all transmembrane mucins, except for MUC4, contains a sea urchin sperm protein enterokinase agrin (SEA) domain, epidermal growth factor-like domains, a transmembrane domain, and a cytoplasmic tail (Lang, 2007; Zaretsky & Wreschner, 2013) (Fig. 1B). Most transmembrane mucins also include one or more EGF domains, except MUC1 and MUC16 (Lang, 2007; Zaretsky & Wreschner, 2013). The C-terminus of MUC4 contains a nidogen-like domain (NIDO), an adhesion-associated domain (AMOP), and VWD domains (van Putten & Strijbis, 2017) (Fig. 1B). The conserved domains in both mucin groups can be used to identify and classify mucin types (Zaretsky & Wreschner, 2013). These two mucin groups have been comprehensively studied in many phyla, but only limited study has occurred in phylum Cnidaria.

Cnidaria is a sister phylum to super phylum Bilateria, but the body plan of cnidarians has only two germ layers (Knack, 2011). Despite their simple body plans, cnidarians can thrive in stressful environments, and many species are particularly common in the intertidal zone. Previous studies on coral species (Phylum: Cnidaria) have reported that mucus has a functional role in protecting the tissue surface (Jatkar et al., 2010). From this study there was an indication that cnidarian mucus may include a range of mucin types, but this has not been examined in detail. This study hypothesised that the sea anemone species, such as *A. tenebrosa* and other cnidarian species have a diverse range of mucin gene families, as these species depend on mucus to survive and protect themselves under the stresses they endure in the intertidal zone. The sea anemone species *A. tenebrosa*, is as an excellent species to investigate the study hypothesis, as it is widespread in Australia, easily identified and collected (Ottaway, 1979; Loh, 2011). Additionally, *A. tenebrosa* produce abundant mucus as they are one of the few anemone groups that are fully exposed during low tide (Fautin, Crowther & Wallace, 2008).

To date, the presence of mucin genes in Cnidaria has been reported for the sea anemone *Nematostella vectensis* (Lang, Hansson & Samuelsson, 2007), and for the coral *Acropora digitifera* (Takeuchi et al., 2016). Although these studies identified the presence of mucin genes in this phylum, they provided a limited view of the mucin repertoire. Furthermore, the study of mucin domain structure in this phylum is almost non-existent and should be investigated to understand the similarity of cnidarian mucin proteins to previously reported mucins in other taxa. Therefore, the current study aimed to identify mucin and mucin-like genes in *A. tenebrosa*, analyse their domain structure and investigate their presence and absence in different cnidarian species. The bioinformatic techniques were selected to identify and analyse new mucin gene sequences. This information will be used to identify the repertoire of the mucin and mucin-like genes in phylum Cnidaria.

## Materials and Methods

### Animal collections

**Figure 2:**
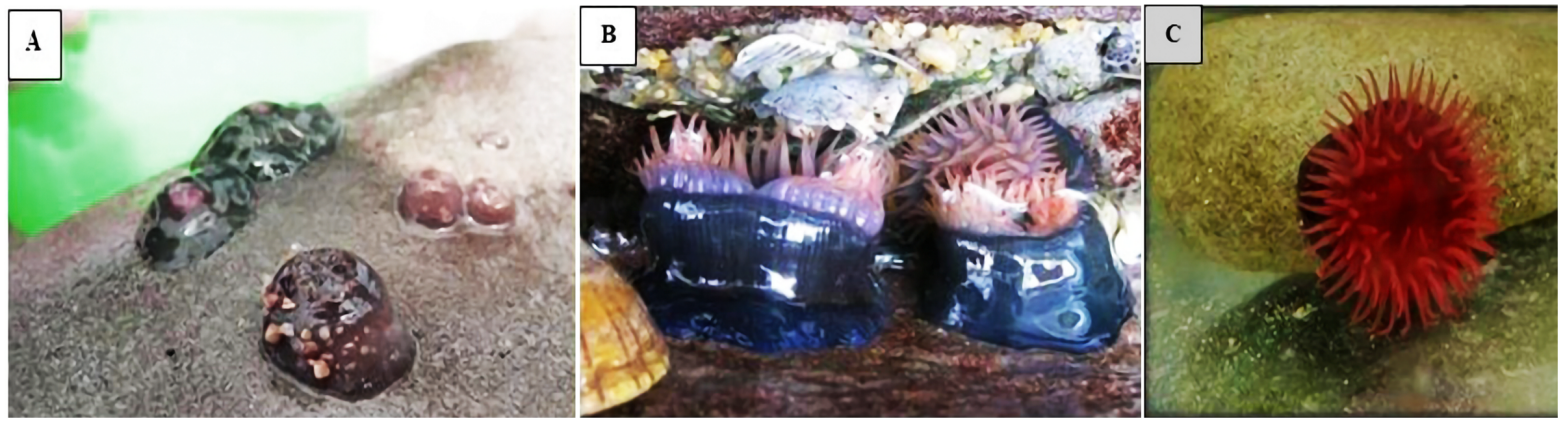
*Actinia tenebrosa* in four colourmorph. The figure shows the sea anemone species *A. tenebrosa* in four colourmorph. (A) Green and brown *A. tenebrosa* at low tide; (B) Blue *A. tenebrosa* fully submerged in water; (C) Red *A. tenebrosa* fully submerged in water (Images by PGL, 2014, 2015).

Four colourmorphs of *A. tenebrosa* (red, green, blue, and brown) were collected during low tide from Point Cartwright, Queensland, Australia (26°32′9.83″S, 153°5′45.12″E) in November 2014 and February 2015 (Fig. 2). Each individual sea anemone was collected and transported to the marine laboratory at the Queensland University of Technology. In this facility, they were housed in 50 L aquarium glass tanks under controlled conditions that reflected their natural environment (see Van Der Burg et al., 2020 for details). Sea anemones were maintained for 1 to 2 weeks, as an acclimation period before RNA extraction was undertaken.

### RNA isolation, sequencing, and quality control

Individual sea anemones were snap frozen in liquid nitrogen and stored at −80 °C until RNA extraction. The individuals were homogenised in liquid nitrogen and total RNA was extracted from the whole organisms using a Trizol/chloroform RNA extraction protocol (van der Burg et al., 2016; Surm et al., 2019). RNA quality and integrity were tested using a bioanalyzer 2,100 RNA nano chip following the protocol of Stewart et al. (2017). Library preparation was undertaken using an Illumina True-Seq stranded mRNA sample preparation kit (Illumina) and following the manufacturer’s instructions. Sequencing was performed on the Illumina NextSeq 500 platform using 150 bp paired end reads. The non-biological sequences as well as low quality reads (Q < 20) were removed using Trimmomatic (Bolger, Lohse & Usadel, 2014; Frischkorn et al., 2014). High-quality sequence reads with thresholds of Q > 20 were used for assembly and other downstream analyses. The raw RNA sequence reads were deposited to GenBank^®^ at the National centre of biotechnology information (NCBI), the accession numbers are available in Table S1

### Assembly, annotation, and Gene Ontology (GO)

The four sets of clean reads (red *n* = 1, green *n* = 1, blue *n* = 1 and brown *n* = 1) were assembled individually using the Trinity v2.0.6 short read *de novo* assembler using default settings and the stranded tag (Grabherr et al., 2011). CD- Hit v.4.6.1 (Li & Godzik, 2006; Fu et al., 2012) was used to remove redundant and chimeric sequences from all assemblies (Stewart et al., 2017). In addition, the core eukaryotic genes mapping approach (CEGMA v.2.5) (Parra, Bradnam & Korf, 2007) was used to assess the assembly completeness by determining the percentage of full-length sequences corresponding to 248 highly conserved eukaryotic proteins (van der Burg et al., 2016). Transcriptome annotation was conducted using the Trinotate pipeline V3.0 Ghaffari et al. (2014), which is available at (https://trinotate.github.io/). Specifically, contigs were annotated using Basic local alignment search tool (BLAST) + v.2.2.31 software (E value 1 × 10^−5^) (Altschul et al., 1990) against the Swiss-Prot and TrEMBL (Uniref90) databases using sequence identity (Suzek et al., 2014). GO terms were assigned to contigs that received BLAST hits and had functional annotation information. The distribution of GO terms across Molecular Function (MF), Biological Process (BP) and Cellular Component (CC) categories were visualised in Web Gene Ontology Annotation Plot (WEGO) (Ye et al., 2006) as per Ali et al. (2015).

### Mucin and mucin-like candidate identification and protein domain analysis

Potential mucin and mucin-like candidates were identified by filtering the annotation results which were generated in Trinotate. Specifically, BLASTx and BLASTp results were filtered using the keywords ‘mucin’ and ‘mucin-like’. This meant sequences with significant BLAST hits to mucin and mucin-like genes in other species were selected as candidate mucin and mucin-like genes. Then, as a validation step, the open reading frames were predicted for the selected mucin and mucin-like sequences using open reading frame finder. Only full-length sequences (presence of start and stop codons), and similar length to homologous mucin proteins were selected. While the partial-length sequences (Absent of start and/or stop codons) were not used in this study.

Domain architectures of the selected mucin and mucin-like sequences were investigated using the Simple Modular Architecture Research Tool (SMART) database (Letunic, Doerks & Bork, 2015) to detect the mucin low complexity regions and determine if candidate mucin proteins included the conserved domains. Detection of low complexity regions and the functional mucin conserved domains helped to confirm mucin sequences and classified them into secreted or transmembrane mucin types.

### Diversity of mucin and mucin-like genes

To investigate the diversity of the selected *A. tenebrosa* mucin and mucin-like candidates in other cnidarians species, *A. tenebrosa* mucin candidates were blasted against other cnidarians species transcriptomes using the local BLAST searches with an E-value of 1 × 10^−5^. The tested cnidarians transcriptomes used in this study included: *Aulactinia veratra*, *Anthopleura buddemeieri*, *Calliactis polypus*, *Nemanthus annamensis*, and *Telmatactis* sp. were generated by ePGL group members at QUT (van der Burg et al., 2016), and as well as other publicly available cnidarian genomic datasets for *Anthopleura elegantissima*, *Aiptasia pallida*, *N. vectensis*, *A. digitifera*, and *Hydra magnipapilatta*. The tested cnidarians species with homologous sequences with the *A. tenebrosa* mucin candidates that contained a mucin domain were included in further analysis.

## Results

### Sequencing and assembly statistical summary

Overall, 152,136,760; 179,309,262; 175,687,690 and 201,995,450 sequence reads were generated from the red, green, blue, and brown colourmorphs, respectively. Reads from the four samples were assembled into 111,882; 105,145; 87,137 and 122,362 contigs for the red, green, blue, and brown colourmorphs, respectively (for more assembly statistic metrics see Table S2). All assemblies were largely complete (>96% for all colourmorphs) and contained a high proportion of full-length transcripts (>92% for all colourmorphs), more statistics are available in Table S2.

### Annotation and Gene ontology

The total number of assembled contigs and significant BLAST hits with a stringency of 1E × 10^−5^ from the red, green, blue, and brown colourmorphs ranged from 64,883 to 46,334 for BLASTx, and from 38,274 to 27,471 for BLASTp. The details are available in Table S3.

GO terms were assigned to 27,172, 23,348, 22,733, and 24,968 contigs from the red, green, blue, and brown colourmorphs respectively. GO terms from each *A. tenebrosa* colourmorph were then individually analysed in WEGO and showed the total assigned gene distribution under each GO category. The highest numbers of GO terms were assigned under BP, followed by MF and CC category. In the BP category, the cellular process and metabolic processes were the most assigned GO terms. Binding and catalytic activity were the top assigned GO terms under the MF category. The greatest number of GO terms assigned under the CC category included: cell, cell part and organelle. WEGO plots was presented in the Figs. S1–S4.

### Identification of mucin and mucin-like candidates and protein domain structure analysis

Based on BLAST and amino acid sequence analyses, the following full-length mucin sequences were identified as transmembrane mucin type including: mucin1-like, mucin4-like, and mucin-like candidates (Table 1). These full-length candidate sequences had multiple isoforms ranging from one to eight for each gene.

**Table 1:** Mucin candidates.

Colourmorphs	Mucin1-like	Mucin4-like	Total identified mucin-like
Red	1	1	3
Green	1	3	2
Blue	1	3	0
Brown	1	3	0

**Note:**

List of full-length identified mucin1-like, mucin4-like and mucin-like candidates generated from the four *A. tenebrosa* colourmorph transcriptomes.

**Figure 3:**
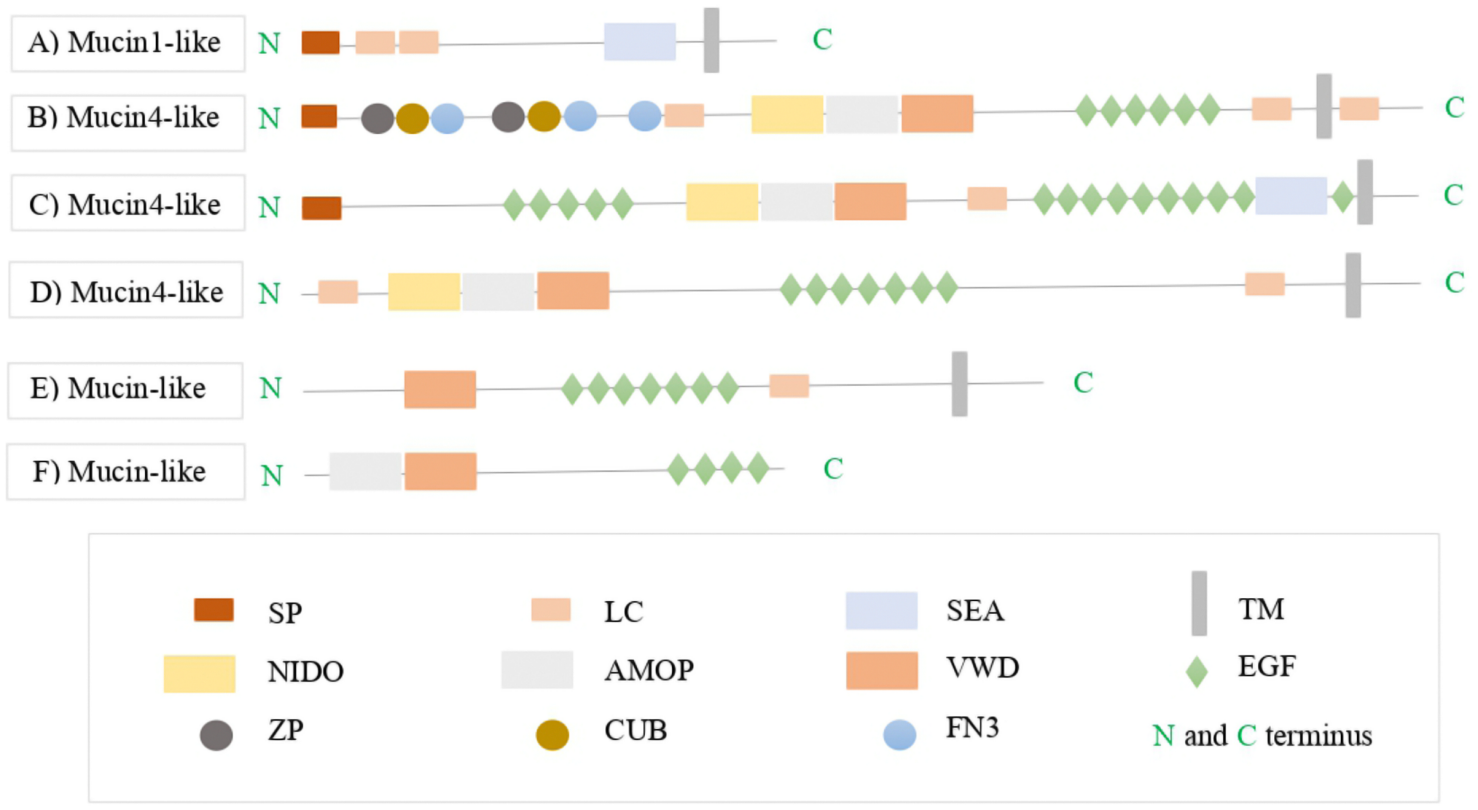
The protein domain architectures of *A. tenebrosa* mucin candidates including domain names. The protein domain architectures of *A. tenebrosa* mucin1-like, mucin4-like and mucin-like candidates. (A) Mucin1-like shows the full protein structure of MUC1 including the N-terminus indicated by SP, one SEA domain followed by transmembrane domain on the C-terminus. (B–D) Mucin4-like sequences show the domain structure of MUC4 which is indicated by the NIDO, AMOP and VWD domains, additional domains on the N-terminus presented in the N-terminus in sequence B, and additional single SEA domain presented in the C-terminus in sequence C. (E and F) Mucin-like candidate structures were lacking in the complete collection of MUC4 domains (NIDO, AMOP and VWD) and so they identified only as mucin-like sequences. Varied numbers and sizes of low complexity regions presented among the candidate sequences. The full definitions of candidate mucins domains are available in Table S8.

The *A. tenebrosa* predicted mucin1-like candidate sequences were annotated as MUC1 (transmembrane-bound type) and found to contain the conserved domain structure of MUC1. These candidates had domain structures that consisted of single SP in the N-terminus followed by two low complexity regions (PTS rich), followed by one SEA domain and one transmembrane domain in the carboxyl C-terminus (Fig. 3A). Since these MUC1 conserved structure were presented its help to identify the candidates as mucin1-like. Top BLASTx and BLASTp hits, domain structures and predicted open reading frame length for this gene from the four *A. tenebrosa* colourmorphs are shown in Tables S4–S7.

Furthermore, the mucin4-like candidates from the four colourmorphs were annotated as MUC4 (transmembrane-bound type). These candidates had domain structures that consisted of NIDO, AMOP, and VWD domains that are part of the conserved architecture of MUC4. The N-terminus and C-terminus of the MUC4 proteins contained a SP and a transmembrane domain, respectively. Some mucin4-like proteins contained additional domains in the N-terminus such as: complement C1r/C1s, Uegf, Bmp1 (CUB), zona pellucida (ZP) and Fibronectin type 3 domain (FN3), while others had an additional SEA domain in the C-terminus (Figs. 3B–3D). The length of the PTS rich low complexity regions among mucin4-like candidates was highly variable and not conserved. The domain organisation of mucin-like candidates lacked in the complete collection of conserved MUC4 domains (NIDO, AMOP and VWD). Therefore, they were identified as mucin-like sequences in downstream analysis (Figs. 3E and 3F). BLASTx and BLASTp hits, Pfam domain structures, and sequence length for this gene from the four *A. tenebrosa* colourmorphs are shown in Tables S4–S7.

In addition to the full-length mucin sequences candidates, four partial-length copies of mucin sequences were also identified from *A. tenebrosa*. These four partial mucin sequences included two gel-forming mucin5B-like sequences, one gel-forming mucin6-like sequence and one transmembrane mucin3A-like sequence. The domain organisation of the partial mucin5B-like genes was conserved across other cnidarian taxa. Mucin6-like and mucin3A-like were too incomplete to investigate their domain organisation or diversity. However, these partial-length mucin sequences were not included as candidates in this study, but their domain structure organisations based on SMART visualisation are available in Fig. S5.

### The diversity of *A. tenebrosa* mucin candidates among cnidarian species Mucin1-like

The mucin1-like gene was identified across most cnidarian species, with exceptions in *A. buddemeieri*, *N. vectensis, Telmatactis sp* and *H. magnipappillata* (Table 2). The mucin1-like protein domain architectures based on SMART visualisation and information generated from blasting *A. tenebrosa* mucin1-like against cnidarian species are available in Table S9 and Fig. S6.

**Table 2:** Mucin candidates diversity.

Cnidarian species	Mucin1-like	Mucin4-like
*A. veratra*	√	√
*A. pallida*	√	√
*A. buddemeieri*	×	√
*P. polypus*	√	√
*N. annamensis*	√	√
*A. elegantissima*	√	√
*Telmatactis sp*	×	√
*N. vectensis*	×	×
*A. digifera*	√	√
*H. magnipapilatta*	×	×
*O. faveolate*	√	√

**Note:**

Table shows the massive diversity of *A. tenebrosa* mucin1-like and mucin4-like candidates across cnidarian tested species. Mucin4-like shows higher presence across the species than mucin1-like.

### Mucin4-like and mucin-like

The mucin4-like gene showed high diversity among cnidarian species examined, except in *N. vectensis*, *H. magnipappillata* and *H. vulgaris* (Table 2). Protein domain architecture (NIDO, AMOP, and VWD) was conserved across most species examined, but the size and position of conserved domains varied among cnidarian species. The N-terminus SP and C-terminus transmembrane domain also varied across the species. The several additional domains that were found in *A. tenebrosa* mucin4-like protein structure including: SEA, CUB, ZP and FN3 were as well detected in other sea anemone tested species. Specifically, the SEA domain was found in the C-terminus of *A. tenebrosa*, was also identified in *N. annamensis, C. polypus* and *A. elegantissima*. While, the additional CUB, ZP, FN3 domains found in the N-terminus of some mucin4-like candidates from *A. tenebrosa*, were also identified in *A. buddemeieri, O. faveolata, A. elegantissima* and *A. pallida*. The mucin4-like protein domain architectures based on SMART visualisation and information generated from blasting *A. tenebrosa* mucin4-like against cnidarian species are available in Table S10 and Fig. S7.

## Discussion

To better understanding of the mucin genes present in *A. tenebrosa* and analyse their structure, current study has used RNA-sequencing, *D. novo* assembly and annotation to identify the mucin and mucin-like genes. Using this approach, we were able to identify in *A. tenebrosa*, a full-length mucin1-like, mucin4-like, and a range of mucin-like genes. The domain structure of the identified mucin genes was found to be like that of the homologous genes in other species, and the majority of the mucin genes were found to be present in the other cnidarian species examined.

### *Actinia tenebrosa* mucin candidates identification and domain structures analysis

Mucin1-like, mucin4-like, and mucin-like were identified in *A. tenebrosa*. The *A. tenebrosa* mucin1-like candidate sequences were found to contain a conserved domain architecture similar to MUC1 (Zaretsky & Wreschner, 2013). Additionally, the mucin4-like candidates had domain structures that consisted of NIDO, AMOP, and VWD domains that are part of the conserved architecture of MUC4 (Zaretsky & Wreschner, 2013). Several additional domains were found in *A. tenebrosa* mucin4-like protein structure including: SEA on the C-terminus, and CUB, ZP and FN3 on the N-terminus. The additional SEA domain needs to be highlighted, as in most previous studies the SEA domain is known to be found in all transmembrane-bound mucins except in MUC4 (Zaretsky & Wreschner, 2013). Zaretsky & Wreschner (2013) proposed that MUC4 originated from a SEA domain-containing ancestor, as did all other transmembrane-bound mucins, but that the SEA domain was lost during evolution. The presence of the SEA domain in cnidarian mucin4-like, could confirm the hypothesis proposed by Zaretsky & Wreschner (2013).

The additional CUB, ZP and FN3 domains on the N-terminus were previously recognised in an extracellular matrix protein of corals (Ramos-Silva et al., 2013; Takeuchi et al., 2016). The role of this protein in *A. tenebrosa*, is still unclear, but they may act as an associated molecule in the mucus layer. The ZP domain is common to several different extracellular proteins, not only mucin. For instance, in *N. vectensis* the ZP domain is found in other extracellular proteins that have roles as a structural component of the oocyte coat and could contribute to the polymerization of the jelly matrix (Levitan et al., 2015). The ZP domain also plays a role during fertilization, preimplantation and oogenesis in mammals (Levitan et al., 2015). This may indicate an important extracellular or reproductive function of the transmembrane-type mucin4-like proteins in some cnidarian species.

Blasting *A. tenebrosa* mucin candidates against other non-redundant protein sequences revealed that several mucin genes found in this species had homologs in vertebrate species. This finding is supported by Miller et al. (2007); Putnam et al. (2007) who reported that sea anemone genomes have a similar gene repertoire to vertebrates. The identification of the full-length transmembrane-mucins, mucin1-like and mucin4-like, in *A. tenebrosa* provides preliminary evidence that the membrane-bound mucins are not restricted to bilaterian taxa. These finding indicate that the diversity of mucins previously reported for phylum Cnidaria (Lang, Hansson & Samuelsson, 2007) was an underestimate.

### *Actinia tenebrosa* mucin candidates diversity among cnidarian species examined

The domain architectures diversity of mucin1-like, mucin4-like and mucin-like gene candidates were present in most other cnidarian species examined (one match from each species). These outcomes provided a better understanding of the diversity of mucins in cnidarians which has previously only been identified in *N. vectensis* (Lang, Hansson & Samuelsson, 2007), and *A. digitifera* (Takeuchi et al., 2016). Since the mucin1-like and mucin4-like were conserved across most tested species, it appears that species from phylum Cnidaria have a diverse repertoire of mucin genes present in their genomes.

## Conclusion

In conclusion, this study carried out an examination of the mucin gene family in cnidarians, with a focus on sea anemones. The *A. tenebrosa* mucin1-like, mucin4-like and mucin-like gene candidates were present in most cnidarian species examined. These outcomes confirmed the study hypothesis that sea anemone species have a diverse repertoire of mucin genes. The analysed mucin sequences architectures were consistent with conserved domains of MUC proteins. Overall, this study has established a baseline for future mucin research in phylum Cnidaria. Future molecular research can extend and build on these outcomes and generate a greater understanding of mucin genes in this phylum.

## Supplemental Information

10.7717/peerj.13292/supp-1 Supplemental Information 1 Supplemental Figures and Tables.

## Additional Information and Declarations

### Data Availability

The following information was supplied regarding data availability: The sequences are available at SRA: SRR3216075, SRR3206038, SRR3207346.
