# Supplementary material for: Identification, diversity and domain structure analysis of mucin and mucin-like genes in sea anemone Actinia tenebrosa
Source: PeerJ. 2022 May 5;10:e13292. doi: 10.7717/peerj.13292 (PMC9080433; doi:10.7717/peerj.13292)
Supplement: Supplemental Information 1 [file peerj-10-13292-s001.pdf]

Identification, diversity and domain structure analysis of mucin and mucin-like genes in sea anemone *Actinia tenebrosa*

Supplementary file:

Sequences read accession numbers, Table 1. Assembly statistics, Table2. Annotations, Table 3. Identification of *A. tenebrous* mucin candidates from (Red, Green, Blue, and Brown colour-morph) Table 4 – Table 7. Mucin domains definitions, Table8. Mucin1-like and Mucin4-like diversity, Table 9, and Table 10. Annotation GO terms using WEGO, Figure 1 – Figure 4. Partial mucins sequences, Figure 5. Mucin1-like domain architectures, Figure 6. Mucin4-like domain architectures, Figure 7.

Supplementary Table 1 | Sequences read accession numbers. The raw RNA sequence reads accession numbers at the National centre of biotechnology information for the four *A. tenebrosa* colour-morph transcriptomes

**Transcriptome**	**SRA accession**
A. tebebrosa (Red colour-morph)	SRR3216075
A. tenebrosa (Green colour-morph)	SRR3206038
A. tenebrosa (Brown colour-morph)	SRR3207346
A. tenebrosa (Blue colour-morph)	SRR3207346

Supplementary Table 2 | Assembly statistics. The assembly statistic data generated from the four *A. tenebrosa* transcriptomes.

**Quality metrics**	**Red**	**Green**	**Blue**	**Brown**
Total reads	152,136,760	179,309,262	175,687,690	201,995,450
Total assembled base pairs	88,116,072	86,177,706	83,348,231	98,585,782
Number of transcripts	111,882	105,145	87,137	122,362
N50	1,478 bp	1609 bp	1,770 bp	1,600 bp
Average contig length	787.58 bp	819.6 bp	956.52 bp	805.69 bp
Maximum length	30,441 bp	30457 bp	31384 bp	32,195 bp
**CEGMA**				
Full length sequences (%)	92.3	96.8	97.98	95.6
CEGs (%)	96.4	98.4	98.79	98.4

Supplementary Table 3 | Annotation results. The table show total number of contigs that received BLASTx and BLASTp hits across the four *A. tenebrosa* colourmorphs

Colourmorphs	BLASTx hit	BLASTp hit
**Red**	46,904	38,274
**Green**	47,370	27,471
**Blue**	46,334	29,250
**Brown**	64,883	37,026

Supplementary Table 4 | Identified mucin1-like, mucin4-like and mucin-like from the red *A. tenebrosa* colourmorph. The table below shows the contig number, blastx and blastp hits, amino acid, and transcript lengths (bp).

Contigs number	Mucin	BLASTx Description	BLASTp Description	Transcript length(bp)	Amino acid length	BLASTx and BLASTp hit
**TR50731|c0_g1**	Mucin1-like	Non	Mucin-1	2040	448	Non<br>MUC1_MESAU
**TR63507|c0_g3**	Mucin4-like	Mucin-like protein	Non	5591	1355	MLP_ACRMI<br>Non
**TR63465|c2_g2**	Mucin-like	Mucin-like protein	Non	6067	962	MLP_ACRMI<br>Non
**TR61953|c2_g3**	Mucin-like	Mucin-like protein	Non	2523	716	MLP_ACRMI<br>Non
**TR63556|c1_g3**	Mucin-like	Mucin-like protein	Non	1234	314	MLP_ACRMI<br>Non

Supplementary Table 5 | Identified mucin1-like, mucin4-like and mucin-like from the green *A. tenebrosa* colourmorph. The table shows the contig number, blastx and blastp hits, amino acid, and transcript lengths (bp).

Contigs number	Mucin	BLASTx Description	BLASTp Description	Transcript length(bp)	Amino acid length	BLASTx and BLASTp hit
**TR49398|c1_g2**	Mucin1-like	Non	Mucin-1	2007	454	Non<br>MUC1_MESAU
**TR43324|c0_g1**	Mucin4-like	Mucin-like protein	Mucin-like protein	7750	1324	MLP_ACRMI<br>MLP_ACRMI
**TR50363|c3_g2**	Mucin4-like	Mucin-like protein	Mucin-like protein	7483	2257	MLP_ACRMI<br>MLP_ACRMI
**TR50175|c7_g4**	Mucin4-like	Mucin-like protein	Mucin-like protein	4762	1345	MLP_ACRMI<br>MLP_ACRMI
**TR50236|c3_g4**	Mucin-like	Mucin-like protein	Mucin-like protein	4688	962	MLP_ACRMI<br>MLP_ACRMI
**TR45099|c4_g13**	Mucin-like	Mucin-like protein	Non	5515	931	MLP_ACRMI<br>Non

Supplementary Table 6 | Identified mucin1-like and mucin4-like from the blue *A. tenebrosa* colourmorph. The table shows the contig number, blastx and blastp hits, amino acid, and transcript lengths (bp).

**Contigs number**	**Mucin**	**BLASTx Description**	**BLASTp Description**	**Transcript length(bp)**	**Amino acid length**	**BLASTx and BLASTp hit**
**TR24970|c9_g2**	Mucin1-like	Non	Mucin-1	1977	454	Non<br>MUC1_MESAU
**TR26748|c2_g1**	Mucin4-like	Mucin-like protein	Mucin-like protein	5873	1332	MLP_ACRMI<br>MLP_ACRMI
**TR24768|c4_g2**	Mucin4-like	Mucin-like protein	Mucin-like protein	5594	1353	MLP_ACRMI<br>MLP_ACRMI
**TR20060|c1_g1**	Mucin4-like	Mucin-like protein	Mucin-like protein	7551	2269	MLP_ACRMI<br>MLP_ACRMI

Supplementary Table 7 | Identified mucin1-like and mucin4-like from the brown *A. tenebrosa* colourmorph. The table shows the contig number, blastx and blastp hits, amino acid, and transcript lengths (bp).

Contigs number	Mucin	BLASTx Description	BLASTp Description	Transcript length(bp)	Amino acid length	BLASTx and BLASTp hit
**TR57482|c19_g3**	Mucin1-like	Non	Mucin-1	2063	454	MUC1_MESAU
**TR56459|c5_g1**	Mucin4-like	Mucin-like protein	Mucin-like protein	7734	1324	MLP_ACRMI<br>MLP_ACRMI
**TR58560|c7_g11**	Mucin4-like	Mucin-like protein	Mucin-like protein	7487	2099	MLP_ACRMI<br>MLP_ACRMI
**TR59496|c2_g1**	Mucin4-like	Mucin-like protein	Mucin-like protein	7470	2268	MLP_ACRMI<br>MLP_ACRMI

Supplementary Table 8 | Mucin domains definition. Table below shows the functional conserved mucin domains found in the structures of *A. tenebrosa* identified mucin1-like and mucin4-like, including the definition for each protein domain.

**Pfam domains**	**Definition**
SEA	Sea urchin sperm protein, Enterokinase, Agrin.
VWD	Von Willebrand factor type D domain
NIDO	Nidogen-like
AMOP	Adhesion-associated domain
TM	Transmembrane helix region/domain
ZP	Zona pellucida domain
CUB	Complement C1r/C1s, Uegf, Bmp1domain
FN3	Fibronectin type 3 domain
EGF	Epidermal growth factor-like domain
LC	Low compositional complexity region
SP	Signal peptide

Supplementary Table 9 | Mucin1-like diversity. This table shows mucin1-like information across cnidarian tested species, generated from blasting *A. tenebrosa* mucin1-like against cnidarian species. It was found that the *A. tenebrosa* mucin1-like presented as a full-length sequence in all cnidarian tested species, with differential in the identical percent, amino acid sequence length and presence of N terminus signal peptide and C terminus domains. The √ refers to presence, while × refers to absence

Cnidarian species	Sequence full length	Identical %	Amino acid length	N-terminus signal peptide	C-terminus SEA domains
A. veratra	√	61.42%	316	×	√
A. pallida	√	43%	387	√	√
C. polypus	√	44.86%	200	×	√
N. annamensis	√	45.23%	200	√	√
A. elegantissima	√	71.69%	321	×	√

A. digitifera	√	33%	449	√	√
O. faveolate	√	36%	548	√	√

Supplementary Table 10 | Mucin4-like diversity. The following table shows mucin4-like information across cnidarian tested species, generated from blasting *A. tenebrosa* mucin4-like against cnidarian species. It was found that the *A. tenebrosa* mucin4-like presented as a full-length sequence in all cnidarian tested species, with differential in the identical percent, amino acid sequence length and presence of N terminus signal peptide and C terminus domain collections. The √ refers to presence, while × refers to absence.

**Cnidarian species**	**Sequence full length**	**Identical %**	**Amino acid length**	**N-terminus signal peptide**	**C-terminus NIDO-AMOP-VWD domains**
A. veratra	√	65.36%	1345	×	√
A. pallida	√	40%	2321	√	√
C. polypus	√	48.89%	1398	×	√
N. annamensis	√	44.42%	2257	√	√
A. elegantissima	√	72.55%	2286	√	√
A. elegantissima	×	74.64%	2164	√	√
A. digitifera	√	40%	1306	×	√
O. faveolate	√	45%	1416	×	√

Telmatactis sp	√	40.52%	1456	√	√
A. buddemeieri	√	66.48%	2171	×	√

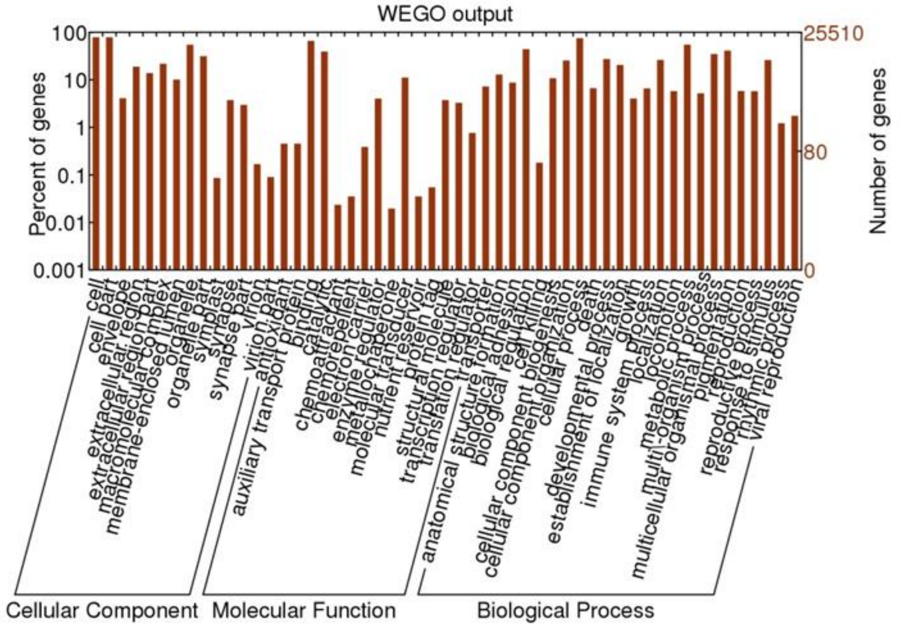

Supplementary Figure 1 | WEGO plots. The distributions of red *A. tenebrosa* gene ontology terms across GO three main categories, Cellular component, Molecular function, and Biological process.

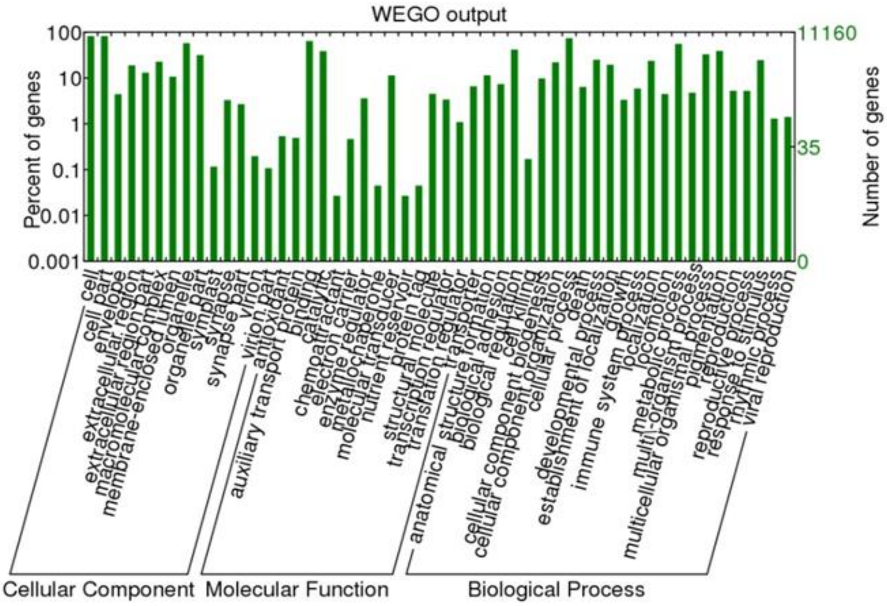

Supplementary Figure 2 | WEGO plots. The distributions of green *A. tenebrosa* gene ontology terms across GO three main categories, Cellular component, Molecular function, and Biological process.

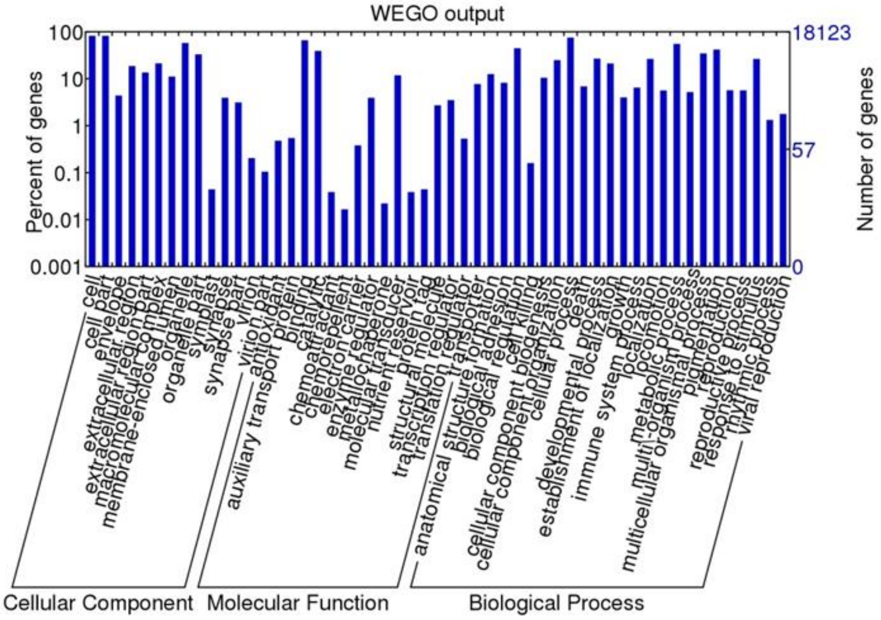

Supplementary Figure 3 | WEGO plots. The distributions of blue *A. tenebrosa* gene ontology terms across GO three main categories, Cellular component, Molecular function, and Biological process.

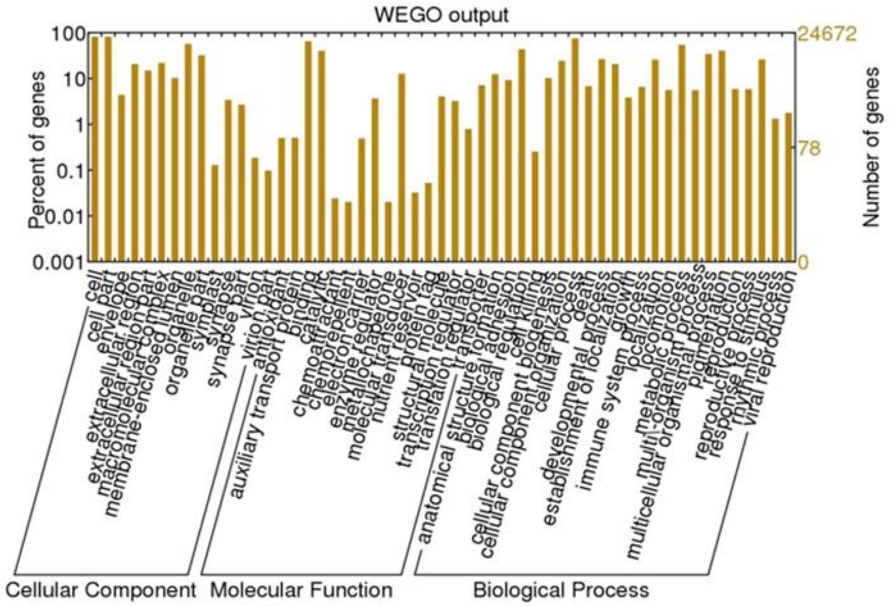

Supplementary Figure 4 | WEGO plots. The distributions of brown *A. tenebrosa* gene ontology terms across GO three main categories, Cellular component, Molecular function, and Biological process.

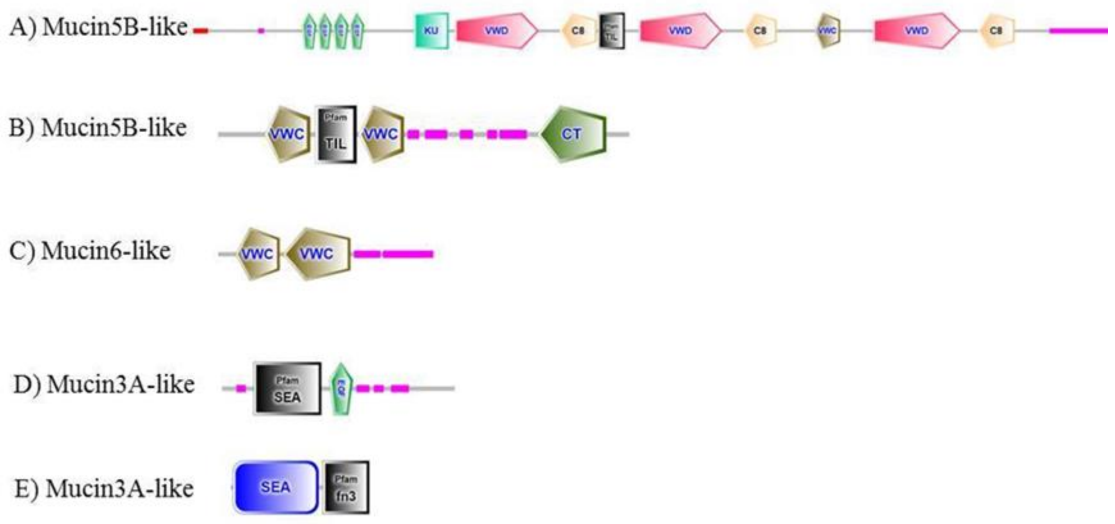

Supplementary Figure 5 | Partial mucins sequences. Figure show the domain structure organisations of the excluded partial identified tow mucin5B-like, mucin6-like and mucin3A-like from the red, green, blue and brown *A. tenebrosa* colourmorphs based on SMART visualisation. A) Partial mucin5B-like structure representing the N-terminus domains and lack of C terminus; B) Partial mucin5B-like structure representing the C-terminus domains and lack of N terminus; C) Partial mucin6-like structure with lack of both N and C terminuses; D) and E) Partial mucin3A-like structures with lack of N and C terminuses.

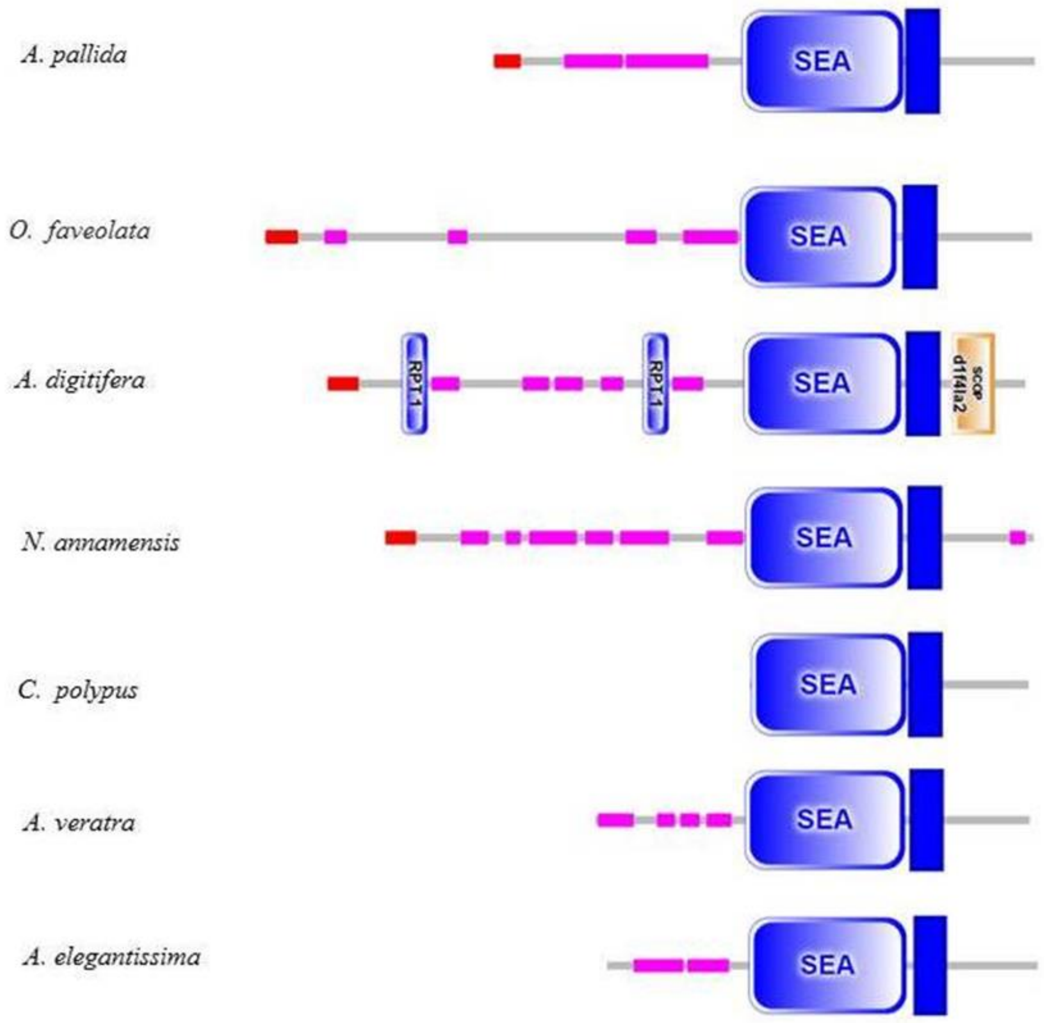

Supplementary Figure 6 | Mucin1-like domain architectures. The figure shows mucin1-like protein domain architectures from the tested cnidarian species based on SMART visualisation. The protein domain structures of MUC1, including a single SEA domain followed by a transmembrane domain on the C-terminus, presented in all tested cnidarian species. The N-terminus indicated by the signal peptide was absent in mucin1-like of some species. The number and size of the low complexity regions were varied among the mucin1-like of cnidarian species.

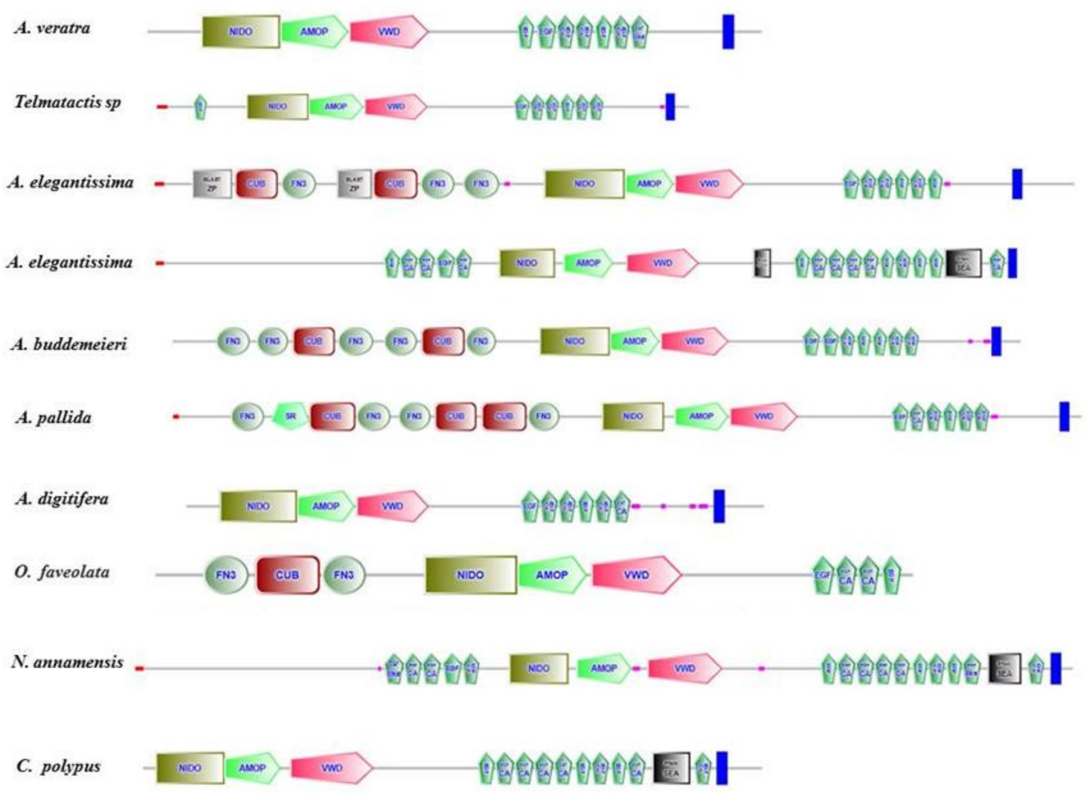

Supplementary Figure 7 | Mucin4-like domain architectures. The figure shows protein domain architectures for mucin4-like genes across the tested cnidarian species based on SMART visualisation. The MUC4 domain structure, including NIDO, AMOP and VWD domains, were presented in most species. *A. tenebrosa* mucin4-like additional domains in the N-terminus were presented only in mucin4-like of *A. elegantissima*, *A. buddemeieri*, *A. pallida* and *O. faveolata*, while the additional single SEA domain in the C-terminus presented only in the C-terminus of *A. elegantissima*, *N. annamensis* and *C. polypus*. The N-terminus indicated by the signal peptide was absent in some sequences.
